# Visitation and stratification of help-seeking youth with mental health problems: a three-year follow-up study

**DOI:** 10.1007/s00127-025-03025-9

**Published:** 2026-03-25

**Authors:** Louise F. Madelaire, Martin K. Rimvall, Pia Jeppesen, Ditte Vassard

**Affiliations:** 1 Department of Child and Adolescent Psychiatry, Copenhagen University Hospital – Psychiatry Region Zealand, Smedegade 16, DK-4000 Roskilde, Denmark; 2 Child and Adolescent Mental Health Center, Mental Health Services – Capital Region of Denmark, Copenhagen, Denmark; 3 Department of Clinical Medicine, Faculty of Health and Medical Sciences, University of Copenhagen, Copenhagen, Denmark

**Keywords:** Child and adolescent mental health problems, Stage-based stepped care, Stratification, Transdiagnostic, Indicated prevention, Municipal setting, Early intervention

## Abstract

**Purpose:**

Stage-based stepped care approaches aim to address gaps between prevention and specialized treatment for mental disorders by directing proportionate interventions in a timely manner. We examined whether increasing stage level, assigned after visitation of youths seeking help in a primary care municipal setting, reflected an increased risk of adverse outcomes after three years.

**Methods:**

Help-seeking youths (6–16 years) were stratified into three stages of developmental psychopathology based on the severity and impact of parent- and self-reported mental health problems corresponding to following levels of need for actions: low-intensity intervention (Stage 1); moderate-intensity preventive intervention, (Stage 2); suggested referral to specialized mental health services (Stage 3). Information on diagnosed mental disorders and prescription of psychotropic medications over the following 3.6 years (median follow-up) was retrieved from the Danish National Registries. Outcomes were compared across the staged groups and compared to an age-matched population-based comparison group (*N* = 16,980, reference group).

**Results:**

Among 566 help-seeking youths, *n* = 74 (13%) were stratified to Stage 1, *n* = 436 (77%) to Stage 2, and *n* = 56 (10%) to Stage 3. Hazard ratios (95% confidence intervals) of receiving a psychiatric diagnosis during three years of follow-up were 3.2 (1.9–5.2) for Stage 1, 3.9 (3.2–4.8) for Stage 2, and 9.0 (6.2–13.3) for Stage 3. Similar stepwise increasing estimates were present regarding use of psychotropic medications, school absence and notifications of concern.

**Conclusion:**

Increased risk of later psychiatric morbidity with increased stage level supports the clinical utility of the stage-based stratification-model for early detection and treatment in the primary care sector.

**Supplementary Information:**

The online version contains supplementary material available at https://doi.org/10.1007/s00127-025-03025-9.

## Introduction

### Background

Globally, nearly one billion people were living with a mental disorder in 2019 – including 14% of the world’s adolescents [1]. The evidence for a worldwide trend of declining mental health in young people is increasingly solid [2]. The ubiquity and scope of this challenge have been confirmed in recent studies [3–5]. In Denmark, a nation-wide study reported a cumulative incidence of 15% for any mental disorder before the age of 18 years in 2016 [6]. However, the number of children (0–17) living with a psychiatric diagnosis has increased by 39% over the last decade, according to a recent national report [7].

Gaps between existing community-based services for mild mental health problems and specialized treatment in Child and Adolescent Mental Healthcare Services (CAMHS) for severe mental health disorders remain a major concern. These gaps in access to services might cause substantial delays in indicated prevention and care for youths who need more help than community counselling, but do not meet the administrative thresholds for referral to specialized CAMHS for diagnosis and treatment [8] – a group referred to as the ‘missing middle’ [9]. Consequently, some youths with milder conditions may experience deterioration of their mental health before receiving appropriate care [10]. A recent study suggests that the duration of untreated symptoms might be especially large for mental disorders with early onset [11], with consequential greater personal suffering and higher societal costs [12].

The identification and interventions aimed at mental health problems in the early stages of the development of psychiatric disorders are key to reducing personal as well as societal burden of mentally ill health [13]. Stepped-care approaches aim to offer interventions of increasing intensity levels, in order to minimize intervention costs and time burden for youths and their families, while still offering evidence-based interventions [14]. However, the stepped-care model has been criticized for potentially delaying appropriate treatment by starting with low-intensity intervention that may be insufficient for some youths [15].

In response to this critique, a stage-based approach to mental health problems and disorders has been suggested to guide early identification and personalized care [16]. This approach allows clinicians to provide more personalized and adaptive care, especially to youths with attenuated syndromes (subthreshold disorders), who have evident need for mental health care that might otherwise go unmet [17]. Where stepped-care focuses on a stepwise increase of intervention intensity and resource allocation, stage-based care emphasizes early identification and intervention at each stage of illness progression. These two approaches can be used complementarily [15], and integrating them aims to direct *proportionate* preventative efforts and treatments to the right group of youths to match their needs in a timely manner.

### Aims

The current study aimed to examine whether increasing stage level, assigned after visitation of youths seeking help in a primary care setting, was associated with a stepwise higher risk of adverse outcomes during three years. To address this aim, we examined register-based outcomes, including mental health service use, over the first three years following a stratification process conducted during the recruitment phase of the Mind My Mind (MMM) randomized controlled trial, which compared a transdiagnostic modular cognitive-behavioral intervention with management as usual [18]. Stage allocation was based on the severity of mental health problems and the corresponding need for care [8]. We therefore hypothesized that higher stage levels would predict an increased risk of adverse outcomes over the following three years.

## Methods

### Study population

Children and adolescents aged 6–16 years with emotional and/or behavioral difficulties were invited to seek assessment in four municipalities in Denmark: Vordingborg, Næstved, Holstebro, and Helsingør. The study participants were assessed consecutively from September 1, 2017, to December 19, 2018, and were stratified into three groups based on the severity of their mental health problems [18]. The study invited youths and their families to sign up for the assessment, which included the evaluation of eligibility for the Transdiagnostic Cognitive Behavioral Therapy Randomized Controlled Trial (MMM RCT). Trial registration (ClinicalTrials.gov Identifier: NCT03535805). Local health and school professionals could provide motivation or assistance to families when needed. Information about the visitation for the MMM trial was published online on school intranets on all public, mandatory schools and municipal websites and distributed to teachers, community-based psychologists, and general practitioners [8]. The help-seeking caregivers made contact with the community-based educational-psychological service via telephone, email, or in-person meeting to sign the youth up for assessment [8]. After returning a written, informed consent to participate in the web-based data collection, the assessment began [8].

Of the 573 help-seeking youths, 566 were assessed and allocated to a stage and thus included in our study population. Furthermore, we included an age-matched comparison group from the background population residing in the four municipalities. Each youth from the visitation cohort of help-seekers was matched 1:30 within the same birth year with comparisons drawn from the background population of 32.830 youths from the four municipalities.

## The stratification process

**Fig. 1:**
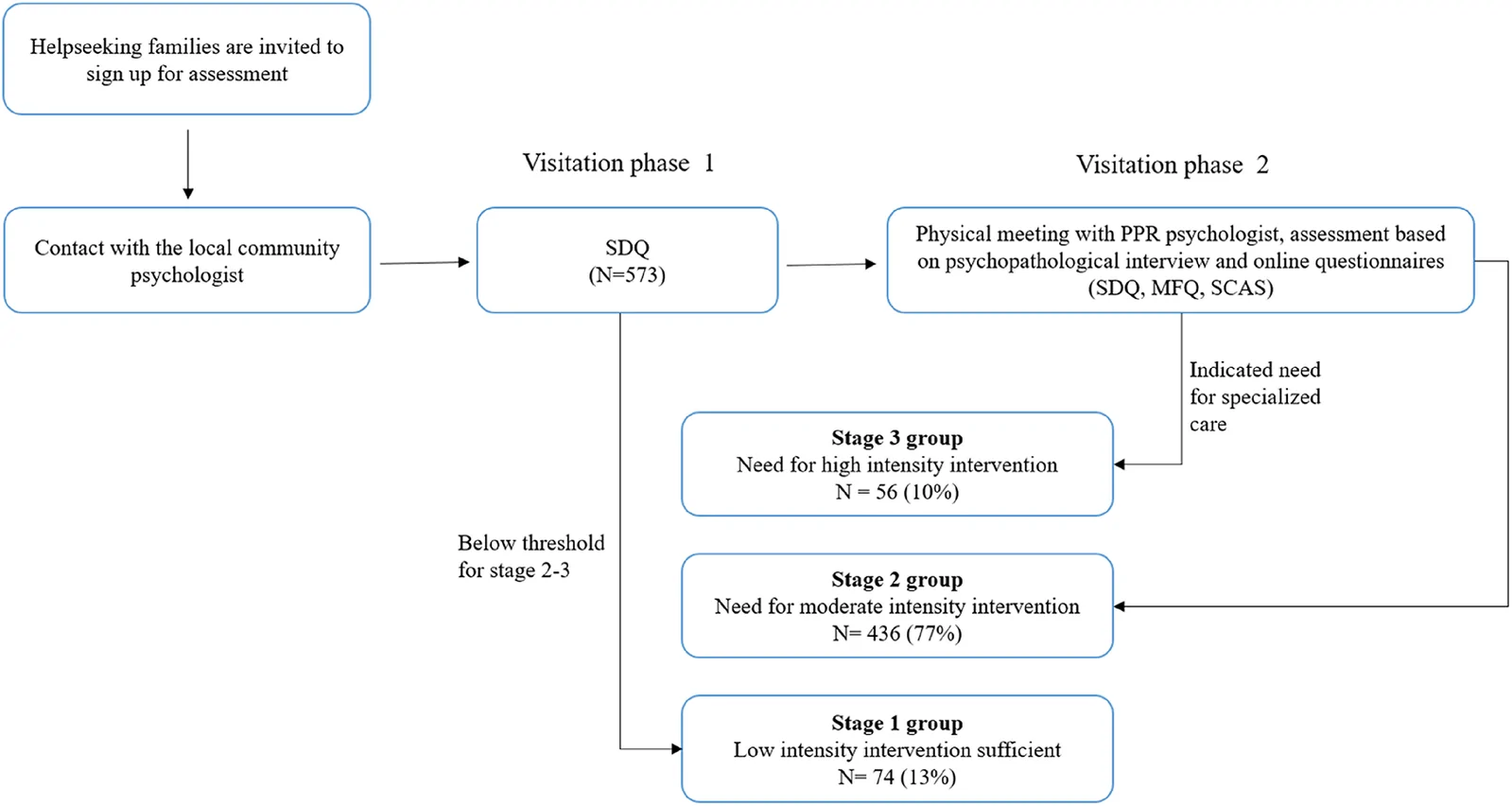
Flowchart of the stratification into stage-based stepped care. Increasing stage level with increasing severity of mental health problems SDQ: Strengths and Difficulties Questionnaire. MFQ: Mood and Feelings Questionnaire. SCAS: Spence Children's Anxiety Scale

The help-seekers were stratified into three levels or stages of developmental psychopathology based on the severity of psychopathology and the impact on daily functioning, see Fig. 1. The aim was to match the population of help-seeking youths to the possible actions [8]:

Stage (1) low intensity intervention: self-help and general counselling.

Stage (2) moderate intensity intervention: individualized psychotherapy or management as usual.

Stage (3) referral to high-intensity, specialized services, e.g. CAMHS.

The stratification procedures consisted of two phases: (1) a web-based initial assessment using the Strengths and Difficulties Questionnaire (SDQ); and (2) a visitation meeting with the psychologist based on standardized methods.

### Phase 1: screening for mental health problems using the strengths and difficulties questionnaire

In the first phase, the help-seeking caregivers completed the SDQ online. The SDQ contains 25 items, covering five subscales related to the youth’s emotional problems, peer problems, behavioral problems, hyperactivity, and pro-social behavior. The first four subscales (scored 0–10) are used to calculate a total difficulties score (0–40) [19]. An impairment score is calculated based on five items, assessing whether the difficulties upset or distress the youth and whether they interfere with home life, friendships, classroom learning, and leisure activities. Each item is scored 0 (no or minor difficulties), 1 (definite difficulties) or 2 (severe difficulties) [20]. SDQ responses that fell above a cut-off algorithm chosen to identify youths with impairing mental health problems (see below) were applied to identify youths with indicated need for early treatment [8]. The lower cut-off was based on age-matched populations in Denmark, Germany and the UK [20–22]. A score above cut-off indicated a mental health state among the 10% of the most affected in these populations [23]: SDQ Impact scores of ≥ 1 combined with at least one of the following scores: total difficulties score of ≥ 14 and/or an emotional problem score of ≥ 5 and/or a behavioral problem score of at least 3 [8]. This cut-off algorithm was effective in identifying youths and adolescents with mental health problems and a high risk of poor school outcomes years later in a general population study [23]. If the parents’ responses to SDQ scored below the cut-off, they were automatically informed that the severity of the youth’s mental health problems were currently on a level where low-intensity intervention, such as self-help and general counselling, was considered sufficient. If the parents reported SDQ scores above the cut-off, they and their youth automatically proceeded to the next phase.

### Phase 2: psychological and psychopathological assessment

In the second phase, youths and parents responded to the Mood and Feelings Questionnaire (MFQ) and Spence Children’s Anxiety Scale (SCAS) online. Subsequently, the family was invited to a meeting with a local community-based psychologist within seven days [8]. The community-based psychologist reviewed the responses from the online questionnaires and conducted a semi-structured psychopathological interview with the youth and parent(s) together. The interview was adapted from the Kiddie Schedule for Affective Disorders and Schizophrenia (KSADS) to capture the main categories of symptoms and impairments above or below the diagnostic threshold. If the assessment revealed signs of (or history of) severe developmental disorder or other severe mental disorder (e.g. intellectual functional impairment, autism spectrum disorder, attention deficit/hyperactivity disorder, psychotic disorder, eating disorder, obsessive–compulsive disorder, repeated self-harm, abuse or dependence of alcohol or psychoactive drugs) the youth was advised to seek referral to CAMHS or other health care professionals for more comprehensive assessment and treatment [4, 10].

### Stage-based stratification

As shown in Fig. 1, 74 youths scored below the SDQ-based threshold for early treatment (Stage 1), 436 youths were found eligible for a moderate intensity intervention, like cognitive behavioral intervention in the MMM trial (Stage 2), while 56 youths were assessed with too severe psychopathology, indicating a need for high intensity interventions, e.g. specialized services in CAMHS (Stage 3). Finally, 6 youths were excluded due to not meeting the age criteria or not completing visitation phase 2 (for details see Fig. 1 in Jeppesen et al.) [18]. The Stage 2 group (*n* = 436) differs from the MMM cohort (*n* = 396), due to a range of exclusion criteria relevant for the RCT, that were not applicable for the present study (e.g. parent/youth did not want to participate in RCT, parent/youth were not able to participate in weekly MMM sessions, main problem not in the domains of anxiety, depressive symptoms, and behavioral problems, and parents did not understand and speak Danish sufficiently to participate in the RCT) [18]. In accordance with this, the Stage 3 group in the present study differs slightly from the Stage 3 group in a previous study by Wolf et al. [23], as the present study also includes individuals with intellectual impairment, severe learning difficulties, or a prior diagnosis of any developmental or mental disorder.

## Register-based measures

The questionnaire and interview data from the stratification process constitute the baseline data in this study, supplemented with information regarding socioeconomic data from the Danish national registers. The use of personal identification numbers across the Danish population-based registers enabled the individual linkage of information. Primarily, we wanted to assess the use of CAMHS in our population by retrieving data from the Danish National Patient Registry, established in 1977 (yet, psychiatric hospitals weren’t included until 1995) [24] and the Danish Prescription Registry, which has recorded information on prescriptions redeemed in Denmark since 1995 [25]. In the Danish National Patient Registry, we were able to retrieve data on diagnoses from admissions and outpatient courses in the public healthcare system, but data from the increasing number of diagnoses administered in the private sector are not accessible. Although by including data from the Danish Prescription Registry, we can extend our knowledge with redeemed prescriptions in our population and thereby we can access information about the proportion of youths who seek specialized treatment (and prescriptions from primary care) in the private sector. Supplement Table 1 lists utilized registers and describes them in more detail.

The primary outcomes were the occurrence of any psychiatric diagnosis and any redeemed prescriptions of psychotropic medicine during follow-up. Regarding the psychiatric diagnosis, we obtained data on public hospital ICD-10 diagnoses. We investigated the differences across the three staged groups regarding the first occurrence of a formal diagnosis in the register [24]. Use of psychotropic medicine was estimated by obtaining data on redeemed prescriptions of psychotropic medicine, and we investigated the differences across the three staged groups regarding the first occurrence of redeemed prescriptions in the register [25].

The secondary outcomes included specific diagnostic categories and specific medicine categories (Supplement Table 2 for more details and specific codes), besides school absence, notifications of concern, parental mental health care use and reduced parental labor market attachment. School absence was investigated as the proportion of youths with school absence above 10% (a commonly used threshold in the Danish School System [26]), 25%, 50% and 75%, respectively, for a cumulated period of three months (not necessarily consecutive). We obtained data on the first occurrence of a notification of concern to social services. Parental mental health care use was estimated by any registration of contact (first registered diagnosis) to the mental health care system or any redeemed prescription of psychotropic medicine after baseline. The overall decline in labor market attachment for both mothers and fathers were measured as the transition from full-time work to part-time/sick leave/unemployment, and the transition from part-time to full-time sick leave/unemployment. All outcomes are listed and described in Suppl. Table 2 and defined as dichotomous variables indicating occurrence/non-occurrence (1/0). The following potential confounders (age, sex, immigration status, household income and mother’s highest educational level) were retrieved from population-based registers (https://www.dst.dk/en/). Immigration status was categorized as “Danish” or “immigrant/descendant of immigrants”. Mother’s highest educational level was classified as “basic school and high school”, “vocational training, short ”, “medium length and bachelor” and “master’s education and above”, and household income as equivalized household income divided into tertiles based on the distribution in the total study population.

### Data analysis

The age-matched background population of youths was used as a reference group for the three staged groups in Cox regression analyses. We primarily reported crude associations and secondarily the analyses adjusted for age, sex, immigration status, household income and mother’s highest educational level. Finally, we report the trend significance across the three staged groups (Stage 1 group, Stage 2 group, and Stage 3 group).

Several sensitivity analyses were conducted. As the youths who participated in the MMM trial were offered intervention immediately after visitation, this was considered a mediating factor potentially influencing the risk of later mental disorder. We addressed this in sensitivity analyses excluding the experimental intervention group at Stage 2. Originally, the help-seekers were invited on the condition that they had no prior mental health diagnosis. However, upon reviewing the registries, we subsequently discovered that some of them were registered with a diagnosis within 18 months prior to baseline. Since the comparison group drawn from the background population in the four municipalities was only matched on age, the criterion of no registered diagnosis within 18 months prior to baseline was applied to the background population in sensitivity analyses to assess whether the applicability of the screening stages was different for a population with no preregistered illness. Time-to-event was examined using Cox proportional hazards regression, with the time point of the initial assessment with SDQ defining the point of entry into the study. We considered and tested for the previously described potential confounders in accordance with our preregistered protocol: https://doi.org/10.17605/OSF.IO/SDHX2. We used a two-sided p-value of 0.05 for all statistical analyses. Results from two regression models are presented as hazard ratios (HRs) with corresponding 95% confidence intervals (CIs) in Table 2. The first model was adjusted for age and gender and the second model was additionally adjusted.

## Results

### Baseline characteristics

Differences in baseline characteristics between the staged groups and the background population are reported in Table 1. We were able to age-match 566 youths to a comparison group of 16,980 youths from the background population in the four municipalities, in size ratio 1:30. The mean SDQ impact score across the three help-seeking groups was 3.9 corresponding to the 90th−94th percentile in the Danish Population [22], and 92% had had a duration of problems of more than 6 months. The main baseline differences between help-seekers and the background population were increased school absence (8% vs. 2% of youths having been absent for at least 25% of school days, respectively) and increased occurrence of notifications of concern (23% vs. 11%) to the municipalities.

**Table 1:** Baseline group characteristics for the matched background population, all staged groups and stage group 1, 2 and 3

	Matched background population 1:30	All staged groups	Stage 1 group	Stage 2 group	Stage 3 group
N (%)	16.980 (100)	566 (100)	74 (13.1)	436 (77.0)	56 (9.9)
Age (5–8), n (%)	4762 (28.0)	158 (27.9)	23 (31.1)	116 (26.6)	29 (33.9)
Age (9–11), n (%)	6867 (40.4)	230 (40.6)	28 (37.8)	185 (42.4)	17 (30.4)
Age (12–16), n (%)	5351 (31.5)	178 (31.5)	23 (31.1)	135 (40.0)	20 (35.7)
Age, mean (SD)	10.7 (2.5)	10.7 (2.5)	10.8 (2.6)	10.7 (2.4)	10.8 (2.9)
Females	8299 (48.9)	267 (47.1)	34 (45.9)	206 (47.3)	23 (41.1)
SDQ imp, mean (± SD)		3.9 (2.2)	1.1 (1.6)	4.2 (1.9)	5.3 (2.3)
SDQ ts, mean (± SD)		16.6 (5.7)	9.6 (4.1)	17.5 (5.1)	19.4 (4.9)
SDQ prosoc mean (± SD)		7.6 (2.1)	8.4 (1.9)	7.6 (2.0)	6.9 (2.4)
SDQ peer, mean (± SD)		2.7 (2.1)	1.6 (1.5)	2.8 (2.1)	3.5 (2.5)
SDQ hyper, mean (± SD)		4.8 (2.8)	3.1 (2.1)	4.9 (2.8)	6.2 (2.3)
SDQ emotion, mean (± SD)		6.4 (2.6)	3.5 (2.0)	6.9 (2.4)	6.3 (2.4)
SDQ conduct, mean (± SD)		2.7 (2.0)	1.4 (1.3)	2.8 (2.0)	3.3 (1.8)
Duration of problems (from SDQ)^1^, n (%)					
> 6 months		503 (92.0)	48 (87.3)	405 (92.9)	50 (89.3)
> 12 months		440 (80.4)	40 (72.3)	355 (64.9)	45 (80.4)
Recent* psychiatric diagnosis, n (%)					
Child	215 (1.3)	NA	NA	5 (1.2)	3 (5.4)
Mother	142 (0.8)	7 (1.2)	NA	NA	NA
Father (*N* = 17.422)	81 (0.5)	4 (0.7)	NA	NA	NA
Psychotropic medicine, n (%)					
Child	193 (1.1)	16 (2.8)	4 (5.3)	8 (1.8)	4 (7.1)
Mother	860 (5.1)	53 (9.4)	5 (6.8)	45 (10.3)	3 (5.4)
Father	555 (3.3)	24 (4.2)	NA	NA	NA
School absence^2^, n (%)					
- School absence < 10%	11,292 (84.8)	330 (60.6)	56 (86.2)	248 (71.5)	26 (61.9)
- School absence 10–25%	1788 (13.4)	88 (19.4)	5 (7.7)	71 (20.5)	12 (28.5)
- School absence > 25%	236 (1.8)	36 (7.9)	4 (6.2)	28 (8.1)	4 (9.5)
Occurrence of recent notification , n (%)					
- Any notification	1916 (11.3)	130 (22.9)	13 (17.3)	96 (22.0)	21 (37.5)
- External notification	382 (2.3)	18 (3.2)	3 (4.1)	11 (2.5)	4 (7.1)
Mother’s labor market attachment^3^, n (%)					
- Self-employed/employed	13,106 (77.8)	443 (78.6)	57 (77.0)	353 (81.2)	33 (60.0)
- Unemployed, on leave, cash benefits	2230 (13.2)	68 (12.1)	10 (13.5)	42 (9.7)	16 (29.1)
- Early pensioner, pensioner	381 (2.3)	23 (4.1)	NA	NA	NA
- Student/other	1129 (6.7)	30 (5.3)	NA	NA	NA
Father’s labor market attachment^4^, n (%)					
- Self-employed/employed	14,434 (87.7)	492 (89.3)	61 (89.7)	388 (90.9)	43 (76.8)
- Unemployed, on leave, cash benefits	1074 (6.5)	33 (6.0)	NA	NA	NA
- Early pensioner, pensioner	459 (2.8)	11 (1.9)	NA	NA	NA
- Student/other	497 (3.0)	15 (2.7)	NA	NA	NA
1st or 2nd generation immigration, n (%)	1692 (10.0)	7 (1.2)	NA	NA	NA
Mother’s highest education^5^, n (%)					
- Basic school and high school	3399 (20.9)	72 (12.7)	10 (14.1)	49 (11.5)	13 (23.6)
- Vocational training, short	6185 (38.0)	220 (39.8)	29 (40.8)	172 (40.3)	19 (34.5)
- Medium length and bachelor	5122 (31.5)	224 (40.5)	26 (36.6)	179 (41.9)	19 (34.5)
- Master’s education and above	1565 (9.6)	37 (6.7)	6 (8.5)	27 (6.3)	4 (7.3)
Father’s highest education^6^, n (%)					
- Basic school and high school	3989 (25.1)	113 (20.9)	11 (16.4)	89 (21.2)	13 (24.1)
- Vocational training, short	7852 (49.4)	282 (52.1)	42 (62.7)	218 (51.9)	22 (40.7)
- Medium length and bachelor	2501 (15.7)	95 (17.6)	9 (13.4)	70 (16.7)	16 (29.6)
- Master’s education and above	1558 (9.8)	51 (9.4)	5 (7.5)	43 (10.2)	3 (5.6)
No. of children in household^7^, n (%)					
1	2851 (16.9)	115 (20.5)	24 (32.4)	81 (18.8)	10 (17.9)
2	8216 (48.7)	302 (53.7)	28 (37.8)	245 (56.7)	29 (51.8)
3+	5810 (34.4)	145 (25.8)	22 (29.7)	106 (24.5)	17 (30.4)
Equivalized household income before tax^7^, n (%)					
1. Tertile	5635 (33.4)	178 (31.7)	23 (31.1)	133 (30.8)	22 (39.3)
2. Tertile	5588 (33.1)	226 (40.2)	32 (43.2)	171 (39.6)	23 (41.1)
3. Tertile	5654 (33.5)	158 (28.1)	19 (25.7)	128 (29.6)	11 (19.6)

*Recent is 18 months before baseline Missing: ^1^ N=29 ^2^ *N* = 3776 ^3^ *N* = 136 ^4^ *N* = 531 ^5^ *N* = 722 ^6^ *N* = 1105 ^7^ N *=* 107

SDQ = Strengths and Difficulties Questionnaire. Unless otherwise indicated, data are expressed as number (percentage) of participants, NA = Not Applicable (observations < 3 in at least one of the groups)

### Follow-up results

**Fig. 2:**
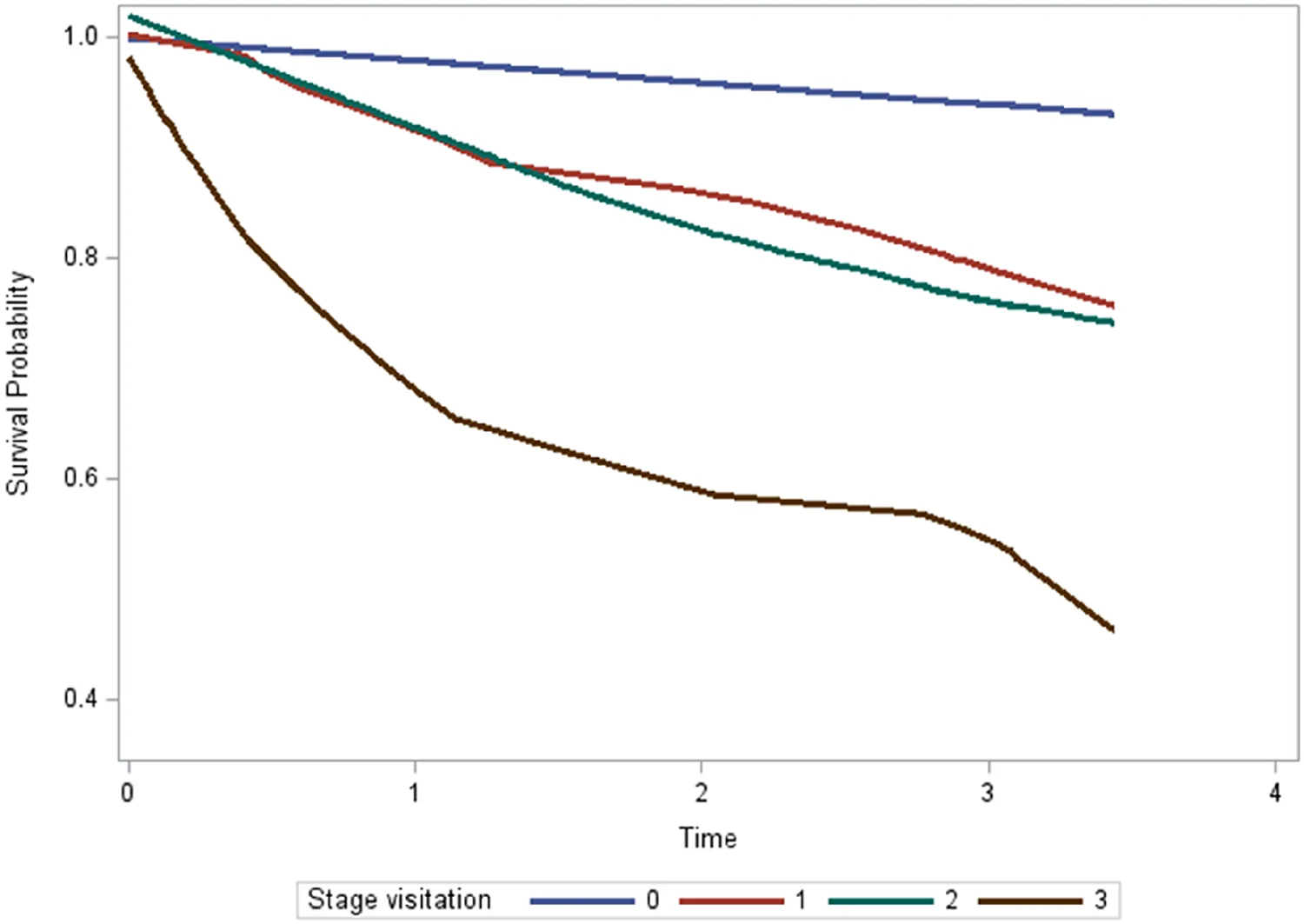
Smoothed Kaplan-Meier Curve of first registered psychiatric diagnoses during follow-up, by stage (in years)

The median follow-up in weeks (10th−90th percentile) was 3.6 years (2.8–4.0) corresponding to 188.6 weeks (145.3–207.4). The proportion of youths who received a psychiatric diagnosis was: 20% (Stage 1), 25% (Stage 2) and 50% (Stage 3), and the proportion was significantly higher in Stage 1 than in the background population (7%) (see Table 2). The risk of being diagnosed with a psychiatric disorder was estimated using adjusted hazard ratios, HR (95% confidence intervals, 95% CI)): stage 1: HR = 3.2, 95% CI 1.9–5.2 (Stage 1), HR = 3.9, 95% CI 3.2–4.8 (Stage 2) and HR = 9.0, 95% CI 6.2–13.3 (Stage 3) (see Table 2 for more details and see Fig. 2 for a graphical representation of the time-to-event analyses of psychiatric diagnoses – model control showed proportionality in hazards). The same pattern applied to redeemed prescriptions for psychotropic medicine: 5% (background population), 12% (Stage 1, HR = 2.6, 95% CI 1.3–4.9), 17% (Stage 2, HR = 3.4, 95% CI 2.7–4.4) and 30% (Stage 3, HR = 5.6, 95% CI 3.4–9.2) (see Table 2). Regarding the specific diagnostic categories the same pattern applied to diagnoses within the affective and schizophrenic spectrum (F20-F48) and for emotional and behavioral disorders with onset usually occurring in childhood (F90-98), however regarding neurodevelopmental diagnoses and prescriptions for ADHD medication and antidepressants, Stage 1 and Stage 2 were similar, however the remaining overall pattern still applied. For the notifications of concern (15% vs. 32% vs. 42% vs. 55%) and school absence > 25% (19% vs. 16% vs. 28% vs. 38%) the pattern of incrementally increased risk with increasing stage applied, except that the background population and Stage 1 were similar regarding school absence. Regarding the parental use of mental health services, there were no clear pattern for mothers and only a weak pattern for fathers (see Table 2).

Test for trends across the three stages (Cochran-Mantel-Haenszel test) were highly significant (p-value < 0.0001) for the primary as well as the secondary outcomes (except mother’s reduction in mental health and reduction in labor market attachment for both parents), indicating that the risk of adverse outcomes were generally increasing with increasing stage. The trend was also highly significant for school absence > 50%, yet the specific estimates could not be reported, since the analyses were based on too few observations in each cell (< 3).

The sensitivity analyses where we (1) excluded the Mind My Mind-intervention group; and (2) excluded the children with recent diagnosis at baseline, did not change the results. We also tested the trend across the stages where we excluded the background population, and still found evidence of the same trends.

**Table 2:** Occurrence and hazard ratios for primary and secondary outcomes over 3 years of follow-up of the staged groups and the matched background population

Outcomes	No visitation *N*=16.980 (100%)	Visitation *N*=566 (100%)				Model adjusted for age and gender (child) HR (95% CI)			Multivariable model* HR (95% CI)		
		All groups *N*=566 (100%)	Stage 1 *N*=74 (100%)	Stage 2 *N*=436 (100%)	Stage 3 *N*=56 (100%)	Stage 1	Stage 2	Stage 3	Stage 1	Stage 2	Stage 3
Primary outcomes											
Any psychiatric diagnosis	1173 (7%)	152 (27%)	15 (20%)	109 (25%)	28 (50%)	3.17 (1.91–5.28)	4.13 (3.39–5.02)	10.47 (7.19–15.23)	3.15 (1.89–5.24)	3.89 (3.19–4.78)	9.04 (6.16–13.27)
ADHD	486 (3%)	57 (10%)	7 (9%)	38 (9%)	12 (21%)	3.22 (1.53–6.79)	3.12 (2.24–4.33)	8.20 (4.62–14.55)	3.31 (1.57–6.98)	2.99 (2.14–4.17)	6.64 (3.64–12.10)
Autism	396 (2%)	64 (11%)	6 (8%)	43 (10%)	15 (27%)	3.82 (1.71–8.57)	4.75 (3.46–6.52)	14.44 (8.61–24.22)	3.82 (1.70–8.56)	4.50 (3.26–6.21)	13.91 (8.28–23.37)
Affective/schizophrenic disorders	468 (3%)	84 (15%)	6 (8%)	64 (15%)	14 (25%)	2.97 (1.33–6.65)	6.19 (4.76–8.04)	11.15 (6.55–18.99)	2.78 (1.24–6.24)	5.80 (4.43–7.58)	10.19 (5.96–17.42)
Emotional and behavioral dis. with childhood onset (F90-98)	383 (2%)	59 (10%)	3 (4%)	44 (10%)	12 (21%)	1.77 (0.57–5.51)	4.69 (3.43–6.40)	10.83 (6.09–19.26)	1.75 (0.56–5.46)	4.44 (3.23–6.11)	9.25 (5.19–16.48)
Use of psychotropic medicine	847 (5%)	101 (18%)	9 (12%)	75 (17%)	17 (30%)	2.54 (1.32–4.91)	3.78 (2.98–4.79)	6.98 (4.32–11.29)	2.56 (1.32–4.93)	3.44 (2.70–4.40)	5.61 (3.42–9.22)
ADHD-medicine	452 (3%)	42 (7%)	6 (8%)	31 (7%)	5 (9%)	2.99 (1.33–6.68)	2.67 (1.86–3.85)	3.44 (1.43–8.29)	3.02 (1.35–6.77)	2.46 (1.70–3.56)	3.03 (1.26–7.34)
Antidepressiva	120 (1%)	22 (4%)	3 (4%)	16 (4%)	3 (5%)	5.52 (1.75–17.40)	5.65 (3.35–9.52)	6.57 (2.08–20.73)	5.14 (1.63–16.25)	5.27 (3.11–8.92)	6.53 (2.04–20.88)
Melatonin	448 (3%)	64 (11%)	4 (5%)	48 (11%)	12 (21%)	2.09 (0.78–5.60)	4.46 (3.31–6.00)	8.86 (4.99–15.73)	2.04 (0.76–5.46)	3.82 (2.80–5.22)	7.66 (4.30–13.65)
Secondary outcomes											
School absence, 3 months above level^1^:											
> 10%	9648 (70%)	396 (82%)	53 (72%)	305 (70%)	38 (68%)	1.25 (0.96–1.64)	1.58 (1.41–1.78)	1.93 (1.40–2.66)	1.26 (0.96–1.67)	1.66 (1.48–1.86)	2.02 (1.47–2.77)
> 25%	2624 (19%)	155 (32%)	12 (16%)	122 (28%)	21 (38%)	0.93 (0.53–1.64)	2.09 (1.74–2.50)	3.48 (2.26–5.34)	1.09 (0.62–1.92)	2.29 (1.90–2.75)	3.64 (2.37–5.59)
> 50%	388 (3%)	40 (8%)	NA	NA	NA	NA	NA	NA	NA	NA	NA
> 75%	161 (1%)	21 (4%)	NA	NA	NA	NA	NA	NA	NA	NA	NA
Notification of concern	2558 (15%)	237 (41.8%)	24 (32%)	182 (42%)	31 (55%)	2.28 (1.51–3.44)	3.54 (3.05–4.12)	5.42 (3.78–7.77)	2.87 (1.90–4.32)	3.76 (3.22–4.40)	6.15 (4.29–8.83)
Parental mental health care use medicine											
Mother	2753 (16%)	151 (27%)	19 (26%)	119 (27%)	13 (23%)	1.68 (1.06–2.67)	1.78 (1.47–2.16)	1.58 (0.91–2.72)	1.68 (1.04–2.71)	1.82 (1.50–2.21)	1.54 (0.89–2.66)
Father^2^	2342 (14%)	87 (16%)	9 (13%)	66 (15%)	12 (21%)	0.82 (0.41–1.63)	1.09 (0.85–1.41)	1.65 (0.94–2.92)	0.78 (0.37–1.63)	1.10 (0.77–1.56)	1.65 (0.94–2.92)
Reduced parental labor market attachment											
Mother	737 (5%)	32 (6%)	NA	NA	NA	NA	NA	NA	NA	NA	NA
Father^3^	504 (3%)	21 (4%)	NA	NA	NA	NA	NA	NA	NA	NA	NA

*Adjusted for immigrant status, household income and mother’s highest educational level, besides age and gender. Number of registrations: ^1^ = 3184, ^2^ = 17.240 ^3^ = 16.033

ADHD= Attention Deficit Hyperactivity Disorder, NA= Not Applicable (observations < 3 in at least one of the groups)

## Discussion

### Main findings

This long-term follow-up of a staging process applied to a large group of help-seeking youths confirmed our hypothesis of an incrementally increased risk of later psychiatric morbidity for each of the three stages compared to the background population. Help-seeking youths were more likely to later use psychiatric services compared to the background population. There was a highly significant trend of increased risk of later psychiatric morbidity with increased stage level. The results were remarkably consistent across a broad range of separate, clinically significant primary and secondary outcomes, based on objective measures drawn from national administrative registries.

### Methodological considerations

A major strength of this study is a thorough and comprehensive questionnaire and interview-based assessment of a large population of help-seeking youths who were stratified into three stages of developmental psychopathology based on this data collection and followed for more than three years by the linkage to individual-level data drawn from several Danish administrative registries, enabling monitoring a wide range of different aspects of social functioning and mental illness. Furthermore, close to complete follow-up of participants was possible due to the use of register-based outcomes, which limited bias due to attrition [27]. Another strength was participation by self-referral, meaning that the study population reasonably represents the help-seeking population in the Danish municipalities. The setup, which involved data originating from four different municipalities in three regions of Denmark increased the generalizability of our findings to the help-seeking population in Denmark and possibly similar Western societies. The ecological validity was high, since the study was conducted in ‘real-world’, primary care-settings. Furthermore, we were able to rule out the impact of participating in the MMM intervention among participants in Stage 2 in our analyses.

Several limitations should be considered when interpreting our findings. First, some relevant information was unavailable in the registries [27]. Youths who were diagnosed in the private sector but without prescribed medications would not be registered. We assume that these cases would be mostly reflected among milder cases, since they were not registered elsewhere. Although this information would have been relevant, it would likely not have diminished our findings. Second, the quality of data in some registers, e.g. regarding school absence, was not optimal for this purpose. School absence was registered as *absence* instead of *presence*, making the interpretation of missing values difficult. We examined the differences in missing values between groups and found no relevant differences. Although a more complete and consistent registration of school absence would have been preferable, the available data appeared to have similar limitations across the staged groups. Third, due to the sample size and the rarity of some mental disorders, grouping of specific diagnoses was required. Fourth, the outcomes regarding diagnoses and treatment only identified the youths who sought and received service provision, missing youths with diagnosable mental health problems who did not seek help. Ultimately, although self-referral can be regarded as a strength, visitation relies on the help-seeking behavior of the families and e.g., youths from immigrant families were less likely to seek help compared to Danish families. Fifth, regarding representativeness of the cohort, the matched background population from the four municipalities had a lower proportion of immigrants (10%) compared to the entire country (around 15%) [28], indicating that the municipalities were not fully representative of immigrants.

Finally, one should also consider that the occurrence of diagnoses and the use of psychotropic medicine is not necessarily an adverse outcome. Youths suffering from mental disorders will likely benefit from being diagnosed in CAMHS, since this often facilitates the possibility of getting help, either in CAMHS, in school, or psychosocial support for the family. These primary outcomes were dependent on the caregivers’ help-seeking behavior and the availability of resources to navigate a complex service system. Hence, we wanted to investigate outcomes that were not dependent on help-seeking behavior or family resources to the same extent. Therefore, in addition to the data from the health registers, we retrieved data on school absence (reported from the schools) and notifications of concern (reported from the municipalities) and the reduction in parents’ labor market attachment (from the Ministry of Employment’s database), to get a more nuanced depiction by including additional objective outcomes.

### Interpretation

While we are not aware of studies utilizing a similar approach to stratification, some studies have examined other stratified care models [29, 30]. The first study [29] demonstrated that stratified care, where patients were allocated to either low- or high-intensity treatments at the initial assessment, corresponding to our stage-based approach, was cost-beneficial compared to stepped-care (in this case, treatment as usual) in a cluster-randomized trial among adults with depression. The second study [30] found that stage 1b level symptoms (corresponding to our stage level 2, in need for indicated prevention) of depression, anxiety and psychosis were interrelated and associated with greater emotional and behavioral difficulties in early adolescence, as well as with life events in late adolescence.

We found that all three stratified groups had increased adverse outcomes compared with the background population. Although youths in Stage 1 presented with only mild symptoms, their use of psychiatric services was markedly higher than that of the background population. It could be speculated that proxies for healthcare use alone may partly reflect help-seeking behavior among caregivers - sometimes referred to as “the worried well” [31]. However, the youths in Stage 1 were also more than twice as likely to have been the subject of a municipal notification of concern as those in the background population during the three-year follow-up. Since this outcome is often independent of parental help-seeking, the “worried well” interpretation is challenged. This suggests an alternative interpretation of this group, e.g. “the prodromal” [31], where the social network has identified a problem not yet detected by the visitation algorithm. This may indicate that our threshold for indicated prevention was set too high.

As for Stage 2, comprising youths with moderate symptoms, we aimed to identify those in need of early, moderate-intensity intervention as indicated prevention. However, as reported previously, 80% of youths at Stage 2 met research-based criteria for mental disorders at baseline [8], and one fourth received a clinical psychiatric diagnosis in CAMHS within three years, based on our results. The most ill individuals in Stage 2, who appeared to present with mental disorders already at baseline, received the full treatment benefits of the MMM intervention [32, 33], however, with no maintenance of effects after three years [34], perhaps calling for a combination of psychotherapy and long-term monitoring with easy access (fast-track) to CAMHS when needed.

For Stage 3, which comprised youths with severe symptoms and a need for specialized services, only half received treatment in CAMHS. There may be multiple reasons for this gap between needs and actual help, e.g. (a) the families had not reached the point where they sought professional help to be referred to CAMHS, (b) did not have resources to comply with appointments, or (c) bureaucratic processes or referral rejection from CAMHS.

We did not observe any parental reduction in labor-market attachment during the follow-up period; however, baseline results showed that only 60% of mothers and 77% of fathers of children in Stage 3 were employed. This may suggest a possible floor effect, where already burdened families had limited labor-market attachment at baseline, making further net reductions during follow-up difficult to capture.

Mental health service providers are experiencing a growing number of help-seeking families. Consequently, the system is becoming increasingly overwhelmed, to the extent that youths in need of effective therapeutic interventions often must wait until their condition deteriorates before receiving the treatment that might have prevented such deterioration. This situation leaves the ‘missing middle’ [9], corresponding to youths at Stage 2, at risk of growing in number while the condition deteriorates.

The stage-based and measurement-based visitation model developed for the MMM trial and evaluated in the current study could, alongside an easily accessible and effective therapeutic intervention implemented within the municipalities, serve as a useful tool in addressing this problem. This approach could be further strengthened by integrating knowledge about which subgroups of help-seekers benefit most from specific forms of indicated prevention.

### Conclusion and future directions

Our hypothesis that an incrementally increased risk of later psychiatric morbidity for each of the three stages was confirmed: the stage steps at baseline cast shadows into the future. The knowledge that we can stratify the help-seeking population according to their subsequent need for service use supports the measurement- and stage-based, stepped care model. However, the relatively high risk of adverse outcome for the help-seeking youths at all levels and the finding that one third of the total group of help-seekers eventually received psychiatric care within three years highlights the need for qualified care at the first levels and easy access to care at the next level – without delay. Thus, early visitation might open an essential window of opportunity to change adverse trajectories for youths with mental health problems.

## Supplementary Information

Below is the link to the electronic supplementary material.

Supplementary Material 1

## Data Availability

The data utilized in the current study are defined as sensitive personal data and cannot be shared publicly due to existing data protection laws in Denmark and imposed by the Danish Data Protection Agency. Further, data from Statistics Denmark is not available to share with unaffiliated parties. In case of interest in working with the data, please inquire with the corresponding author.
