# Supplementary material for: Visitation and stratification of help-seeking youth with mental health problems: a three-year follow-up study
Source: Soc Psychiatry Psychiatr Epidemiol. 2026 Mar 25;61(9):1833–44. doi: 10.1007/s00127-025-03025-9 (PMC13578050; doi:10.1007/s00127-025-03025-9)
Supplement: Supplementary file 1 [file 127_2025_3025_MOESM1_ESM.docx]

| **Supplementary Table 1. Overview of registers** | | | | |
| --- | --- | --- | --- | --- |
| **No.** | **Register** | **Establishment*** | **Description (Period of follow-up)** | **Variable of interest** |
| 1 | The Danish National Patient Registry | 1977  (yet, psychiatric hospitals weren’t included until 1995) | Contains data from public hospitals and patient courses, including diagnoses. Originates from the Danish Health Agency (SDS: Sundhedsdatastyrelsen)  (Follow-up from entry date to September 17, 2021) | ICD10-diagnoses  (F10-F99) |
| 2 | The Danish Prescription Registry | 1995 | Contains data from redeemed medicine prescriptions from both public and private sector. Originates from SDS  (Follow-up from entry date to June 30, 2021) | Redeemed prescriptions of psychotropic medicine |
| 3 | FAIK  (Part of the Basic Database of Statistics Denmark) | 1987 | Contain data on family income  (Baseline data) | Equivalent family income |
| 4 | AKM  (Labor market classification. Part of the Basic Database of Statistics Denmark) | 1991 | Contains data on employment courses. Originates from the Ministry of Employment.  (Follow-up from entry date to October 31, 2021) | Change in labor market attachment |
| 5 | School absence  (Ministry of Children and Education) | 2010/2011 | External registry. Contains information on children’s school absence. Originates from the Ministry of Children and Education’s data warehouse. School absence reported by the individual class teachers.  (Follow-up from entry date to June 30, 2021) | School absence |
| 6 | BUU  (Statistics Denmark) | 2015 | Data reported from the municipalities.  (Follow-up from entry date to December 31, 2020) | Notifications of concern |
| **Abbreviations:** AKM=Arbejdsklassifikationsodulet BUU=Børn og Unge – Underretninger (Danish) FAIK=Familieindkomster (Danish) SDS=Sundhedsdatastyrelsen (Danish)  * Statistis Denmark ( <https://www.dst.dk/extranet/forskningvariabellister/Oversigt%20over%20registre.html>) and Ministry of Child and Education (<https://www.uvm.dk/statistik/grundskolen/elever/elevfravaer> | | | | |
|  | | | | |

| **Supplementary Table 1. Overview of registers** | | |
| --- | --- | --- |
| **Register-based measures** | **Data source** | **Variable description** |
| Psychiatric diagnoses  (Primary outcome) | The Danish National Patient Registry | We will obtain data on public hospital ICD-10 diagnoses and investigate the differences across the three staged groups regarding the occurrence of receiving a formal diagnosis or not. According to a transdiagnostic dimensional approach, and in line with the follow-up study of the RCT, we will use broad categories of ICD-10 diagnoses:  (1) Any psychiatric diagnosis (F10-99)  (2) Anxiety, affective and schizophrenia spectrum disorders (F20-F48, the three diagnostic categories are collapsed into one transdiagnostic group of disorders with episodic or recurrent symptoms)  (3) Autism spectrum disorders (F84-F89)  (4) Hyperkinetic and behavioral disorders (F90-F91)  (5) Behavioral/emotional disorders with childhood onset (F92-F99) |
| Psychotropic medicine  (Primary outcome) | The Danish National Prescription Registry | Use of psychotropic medicine will be estimated by obtaining data on predeemed prescriptions on psychotropic medicine. By doing so, we will also detect some of the children and adolescents who have received prescriptions for psychotropic medicine in the private sector, which are not included in the Danish National Patient Register. We will investigate the differences in the proportion of youths who were prescribed psychotropic medicine across the three stages. We will use the following medicine categories:  (6) Any psychotropic medicine  (7) Antipsychotic medication (N05A, and subcategories)  (8) ADHD medication (N06B, N06AX12, C02AC02, and subcategories)  (9) Antidepressants (N06A, and subcategories)  (10) Anxiolytics, hypnotics (N05BA, N03AE, N05CF, and subcategories)  (11) Melatonin |
| School absence | Registry from the Ministry of Children and education | We will investigate how many days the child has been absent for each month (out of the amount of active school days) and compare the occurrence of school absence across the three staged groups. We will look at it as:  (12) School absence as a dichotomous outcome where we investigate the proportion of youths with school absence above 10% of schooldays, which is a commonly used threshold in the Danish School System. Furthermore, we will explore school absence of above thresholds of 25%, 50% and 75% for a cumulated period of 3 months (not necessarily consecutive). |
| Notifications of concern to the municipality | BUU  (Children and adolescents) | For the outcome of notifications of concern to social services, it will not be possible to distinguish between sources of the concern on a detailed level. Registrations are often co-occurring or repeated with several sources at the same time, reflecting that, the notifications are also used as an administrative tool when expressing the need for an intervention in the municipality. We will, however, specify some external sources of concern: police, court, crisis center or anonymous, which could indicate a more acute concern. Notifications are therefore included as  (13) The occurrence of any notification  (14) The occurrence of concern from these specific mentioned sources. |
| Parental mental health care use | The Danish National, The Danish National Prescription Registry | Estimated by occurrence of mental disorder or use of psychotropic medicine in parents.  (15) Any registered diagnosis to the mental health care system or predeemed prescription of psychotropic medicine |
| Reduced parental labor market attachment | AKM (labor market classification) | (16) The overall decline of labor market attachment for both mother and father, measured as the transition from full time work to part time/sick leave/unemployment, and the transition from part time work to full time sick leave/unemployment. |
| **Abbreviations** AKM=Arbejdsklassifikationsodulet BUU=Børn og Unge – Underretninger (Danish) | | |
